# Potential public health benefits from cat eradications on islands

**DOI:** 10.1371/journal.pntd.0007040

**Published:** 2019-02-14

**Authors:** Luz A. de Wit, Donald A. Croll, Bernie Tershy, Dolores Correa, Hector Luna-Pasten, Paulo Quadri, A. Marm Kilpatrick

**Affiliations:** 1 Department of Ecology and Evolutionary Biology, University of California Santa Cruz, Santa Cruz, California, United States of America; 2 Laboratorio de Inmunología Experimental, Instituto Nacional de Pediatría, Ciudad de México, México; 3 Department of Environmental Studies, University of California Santa Cruz, Santa Cruz, California, United States of America; University of California Berkeley, UNITED STATES

## Abstract

Cats (*Felis catus*) are reservoirs of several pathogens that affect humans, including *Toxoplasma gondii*. Infection of pregnant women with *T*. *gondii* can cause ocular and neurological lesions in newborns, and congenital toxoplasmosis has been associated with schizophrenia, epilepsy, movement disorders, and Alzheimer’s disease. We compared seroprevalence of *T*. *gondii* and risk factors in people on seven islands in Mexico with and without introduced cats to determine the effect of cat eradication and cat density on exposure to *T*. *gondii*. Seroprevalence was zero on an island that never had cats and 1.8% on an island where cats were eradicated in 2000. Seroprevalence was significantly higher (12–26%) on the five islands with cats, yet it did not increase across a five-fold range of cat density. Having cats near households, being male and spending time on the mainland were significant risk factors for *T*. *gondii* seroprevalence among individuals, whereas eating shellfish was protective. Our results suggest that cats are an important source of *T*. *gondii* on islands, and eradicating, but not controlling, introduced cats from islands could benefit human health.

## Author summary

Infection with *T*. *gondii* can cause miscarriage or severe ocular and neurological lesions in newborns, systemic disease in immunocompromised individuals, and has been linked to mental disorders and neurodegenerative diseases such as schizophrenia, Alzheimer’s and movement disorders in adults. On the majority of islands, introduced cats are the only species capable of maintaining the sexual phase of the life cycle of *Toxoplasma gondii*. Introduced cats on islands are also responsible for 14% of all bird, mammal and reptile extinctions. Their management, which has been implemented as a biodiversity conservation measure, has the potential to reduce or eliminate the burden of diseases caused by *T*. *gondii* in island communities via control of its definitive host. To examine if management of introduced cats could reduce risk of infection with *T*. *gondii*, we compared the seroprevalence and risk factors associated with *T*. *gondii* exposure in people on seven islands with variation in cat density, including one island in which cats were eradicated in the year 2000, and another island in which cats had never been present. We found that eradication of introduced cats on islands could significantly reduce human risk of exposure to *T*. *gondii*. Seroprevalence of *T*. *gondii* was zero on the island that never had cats and near zero on the island where cats were eradicated. Furthermore, all island resident children born after cats were eradicated showed no evidence of exposure to the parasite. The odds of seropositivity to *T*. *gondii* were nearly five-fold higher in people that had cats near their homes. On islands with cats, we found no association between local cat density and *T*. *gondii* seroprevalence, suggesting that complete eradication rather than control of cat population densities is necessary to reduce public health impacts of toxoplasmosis.

## Introduction

Cats (*Felis catus*) are reservoirs of many pathogens that affect humans, including the parasite *Toxoplasma gondii* [1]. Cats are also the second most widespread introduced predator found on islands [2,3], and have contributed to 14% of global bird, reptile and mammal extinctions on islands [2]. The dual impact of introduced cats on wildlife and human health increases the potential benefits of eradicating cats from islands [4,5]. Introduced cats have been eradicated for conservation reasons from 80 islands globally [6], resulting in rapid recoveries of native species on many of those islands [3]. Fifteen of these cat eradications were on islands with permanent human settlements [6]. A key gap in our knowledge is whether eradication or control of introduced species also result in public health benefits.

Toxoplasmosis is one of the most widespread zoonotic diseases with a significantly greater burden in low-income countries, and cats are a key reservoir host [7]. Domestic cats and wild felids are the only known definitive hosts for *T*. *gondii* [8]. Cats can become infected after ingesting *T*. *gondii* bradyzoites found in tissue cysts of infected intermediate hosts (i.e. prey such as rodents or birds)[9]. Acutely infected cats host the sexual cycle of the parasite and subsequently shed millions of *T*. *gondii* oocysts in their feces [9–11], thereby contaminating the soil or bodies of water [12]. Oocysts sporulate in the environment and become infectious to intermediate hosts and people [8]. The burden of toxoplasmosis tends to be highest in low-income countries from tropical regions, with prevalence rates ranging between 35.8% and 85.4% [13–20]. Women exposed to *T*. *gondii* during pregnancy can transmit the parasite to their fetus, which can lead to miscarriage or congenital toxoplasmosis [7]. Congenital toxoplasmosis can result in severe ocular and neurological lesions in newborns [21–23] and has been linked to schizophrenia, epilepsy, movement disorders and Alzheimer’s disease [24,25]. Furthermore, *T*. *gondii* infection can be acquired postnatally leading to vision loss [23] and systemic disease in immunocompromised individuals [26]. There is currently no vaccine against *T*. *gondii* and treatment is commonly restricted to acute infections, particularly for women infected during pregnancy or immunosuppressed patients [10,21].

Most islands do not have native felid species [27], creating the potential to reduce the burden of *T*. *gondii* infection in people living on islands by reducing or eliminating introduced cat populations. Local sources of *T*. *gondii* on islands may include contact with shedding cats, oocyst-contaminated soil or consumption of local shellfish that have been contaminated by runoff that carries *T*. *gondii* oocysts from land to sea [28,29]. External sources of infection include consumption of contaminated meat and vegetable products that are imported from the mainland, and exposure during travel to a region where *T*. *gondii* is endemic.

**Fig 1:**
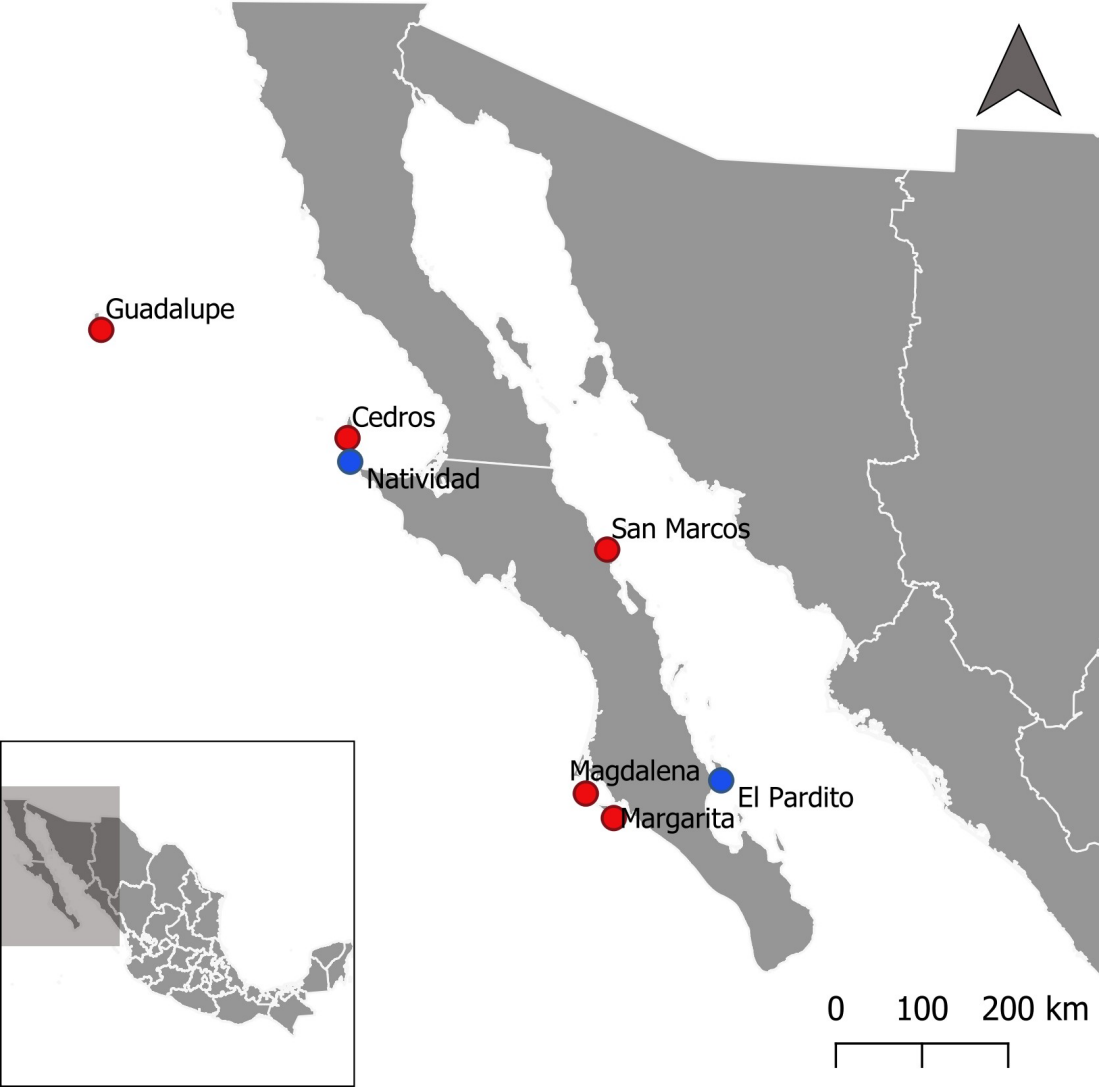
Geographic distribution of the seven human inhabited islands of Baja California, Mexico. Islands where cats are absent are color-coded blue; islands where cats are present are color-coded red.

Although there have been many studies that attempt to correlate prevalence of *T*. *gondii* in soil, or *T*. *gondii* seroprevalence in pigs, humans, rodents, or cats with some measure of cat abundance or exposure [30–39], only one of these studies [32] estimated cat density using a standardized approach, and none attempted to determine the quantitative relationship between cat density and *T*. *gondii* exposure in humans. This relationship is needed to determine how low cat density must be reduced to achieve a reduction in *T*. *gondii* exposure in humans. Thus, our goal was to determine if reducing or eliminating populations of introduced cats could reduce *T*. *gondii* exposure in human populations on islands. We examined risk factors and the seroprevalence of *T*. *gondii* exposure in people on seven human inhabited islands located off the coast of the Baja California Peninsula, Mexico (Fig 1). These islands do not harbor native felids and have a range of introduced cat densities, including one where cats have never been present and another where cats were present but eradicated in 2000.

## Methods

We conducted research on seven islands in Baja California, Mexico (Fig 1), between February 2016 and October 2017. The human communities on these islands rely on fishing as their main source of protein and income, except for San Marcos, which relies mostly on gypsum mining. The climate is tropical and subtropical desert with high annual mean temperatures (19.5–23.7°C), and low precipitation (47.6–281.19 mm/year) [40]. All islands except Natividad and El Pardito had populations of introduced cats at the time of our study. El Pardito is a small island with a fishing community of 13 people and has never had cats. Cats were introduced to Natividad in the 1920s, and fluctuated over time until they were eradicated in 2000 [41,42]. Cats on the rest of the islands live mostly as free-roaming (57% of the participants report occasionally feeding food scraps to cats) or feral (38% of participants do not report feeding cats), and a small fraction of cats are owned (6% of participants report buying cat food and regularly feed cats) but all are allowed to go outdoors. Cat populations may have decreased on Guadalupe and Magdalena Islands over the past decade due to control efforts [43], but no data on cat abundance over time in the human inhabited regions of the islands is available.

### Sample collection

We systematically visited households and we visited community aggregation centers (i.e. schools, convenience stores, administrative offices of the fish cooperatives and a gypsum mine), where we distributed information pamphlets about the study. To determine *T*. *gondii* seropositivity and examine possible risk factors we collected blood samples and applied a questionnaire to people who had given informed consent, and in the case of underage children, if they were accompanied by their parent or tutor and had given informed consent.

### Seroprevalence estimates

We used the fingerprick method to collect approximately 10 μL of blood on Guthrie cards (filter paper) and refrigerated cards at 4°C until we analyzed them at the *Laboratorio de Inmunología Experimental* of the *Instituto Nacional de Pediatría*, *México*. We tested all samples for presence of IgG antibodies against *T*. *gondii* using an indirect ELISA [44]. We determined the cutoff value for seropositivity in each ELISA run as the average optical absorbance of the negative controls plus three standard deviations of the absorbance from negative samples [45]. We ran each sample in duplicate and considered it as positive if the average optical absorbance was greater than the cutoff value.

We estimated crude *T*. *gondii* antibody prevalence and created age-adjusted estimates using the direct method [46] with the age-structure of the 2015 population of the States of Baja California and Baja California Sur, Mexico [47].

### Questionnaire

To examine associations between risk factors and *T*. *gondii* exposure, we interviewed participants using a standardized questionnaire [48,49] adapted to the social context of communities in the islands of Baja California (S1 Appendix). For each individual we recorded gender; age; educational level; source of drinking water; whether they had contact with soil through outdoor activities; consumption of raw or undercooked meat or poultry; annual frequency of meat (including poultry and pork), and shellfish consumption; annual frequency of travel outside the island; and fraction of time spent outside the island. We asked people if they had cats; whether cats were allowed indoor, outdoor, or both; if they were in contact with cat feces when cleaning their household; and we asked them to estimate the number of cats observed near their house. To better understand cat ownership and the relationship between people and cats in the islands, we asked people if they fed the cats that roamed near their household and whether they fed them food scraps or cat food. We also asked people whether they owned a dog, and if it was allowed indoor, outdoor, or both, as dogs may act as carriers of *T*. *gondii* oocysts in their fur [50]. We excluded water as a potential source of *T*. *gondii* exposure because all islands obtain water from local desalination plants or from fishing boats with desalination equipment (El Pardito), from which water is delivered to each household through water pipes or barrels.

We interviewed and collected blood samples from all 13 residents on El Pardito, and 59–325 participants on each of the six remaining islands (representing 25–75% of each population), for a total of 724 participants of ages 9 to 70 (Table 1 and S2 Appendix).

**Table 1:** Estimated crude and age-adjusted *Toxoplasma gondii* seroprevalence and odds ratio in the islands of Baja California, Mexico.

Island	% Crude seroprevalence (no. positive / no. sampled)	% Age-adjusted seroprevalence (95%CI)	odds-ratio relative to Natividad (95% CI)
**El Pardito**	0 (0/13)	0 (0–0)	ND
**Natividad**	2.13 (2/94)	1.76 (1.75–1.77)	1
**Cedros**	13.84 (45/325)	11.62 (11.59–11.65)	7.4 (2.2–45.8) *
**San Marcos**	25 (25/100)	22.89 (22.85–22.94)	15.3 (4.4–97) **
**Margarita**	25.3 (19/75)	16.61 (16.58–16.66)	15.6 (4.3–100.3) **
**Magdalena**	27.11 (16/59)	25.03 (24.98–25.1)	17.1 (4.6–111.4) **
**Guadalupe**	27.6 (16/58)	25.68 (25.64–25.73)	17.5 (4.7–114.1) **

CI = Confidence Interval; ND = not defined;

* P < 0.01;

** *P <* 0.001

### Cat density

We used distance sampling along transects (178–433 meters in length) on each island to measure cat density (S3 Appendix) [51]. We placed transects in human-occupied areas, including main roads in towns. We walked transects between 30 and 90 minutes after dawn on each of two consecutive days, counted all cats, and estimated the distance to each cat with the aid of a rangefinder (Bushnell Yardage Pro Sport 450). We used the ds function in the *Distance* package in R [52,53] to estimate cat density on each island. We selected the best fitting detection function on each island using Akaike’s Information Criterion with correction for small sample sizes (AICc) and used the Cràmer-von Mises test to assess the goodness of fit of the best fitting function [53] (S3 Appendix). To examine the relationship between *T*. *gondii* seroprevalence and cat density among islands, we fit a nonlinear saturating function (Seroprevalence = Y_int_+c_0_*(1-e^(-c1*Cat Density)^) to the data with a binomial distribution using the mle2 function in the *bbmle* package in R. In this model, Y_int_ is the seroprevalence when cat density is zero, (c_0_+Yint) is the asymptote (the seroprevalence at high cat densities), and c_1_ is the slope parameter describing the increase in seroprevalence with cat density.

### Statistical analysis

We used R version 3.3.3 to run all statistical analysis [52]. We used logistic regression models to compare age-adjusted seroprevalence among islands. We used Fisher’s Exact Tests to compare seroprevalence among islands for children born after 2000, when cat eradication took place in Natividad. For the risk factor analysis, the predictor variables associated to cats were correlated (r>0.3). To avoid including many collinear variables, we ran two generalized linear mixed effects models (with a binomial distribution and a logit link) including data from all the islands, with island as a random effect. In the first model, we included the presence/absence of cats reported near households, and all non-cat related predictor variables, but removed all other variables related to cats. We then fit a second model for the subset of people that reported having cats near their households that included all non-cat related variables, exposure to cat feces and the number of cats a person reported having near their house.

### Ethics statement

All research was performed under the human subjects protocols CONBIOETICA02CEI00520131206 and CONBIOETICA03CEI00120131203 approved by the Human Subjects Research review committees of the State of Baja California and Baja California Sur, Mexico, and protocol HS2385 approved by the Office of Research Compliance Administration of the University of California Santa Cruz. All adult subjects provided written informed consent, and in the case of underage children who participated in the study, a parent or guardian provided written informed consent on the child’s behalf.

## Results

### *T*. *gondii* seroprevalence

**Fig 2:**
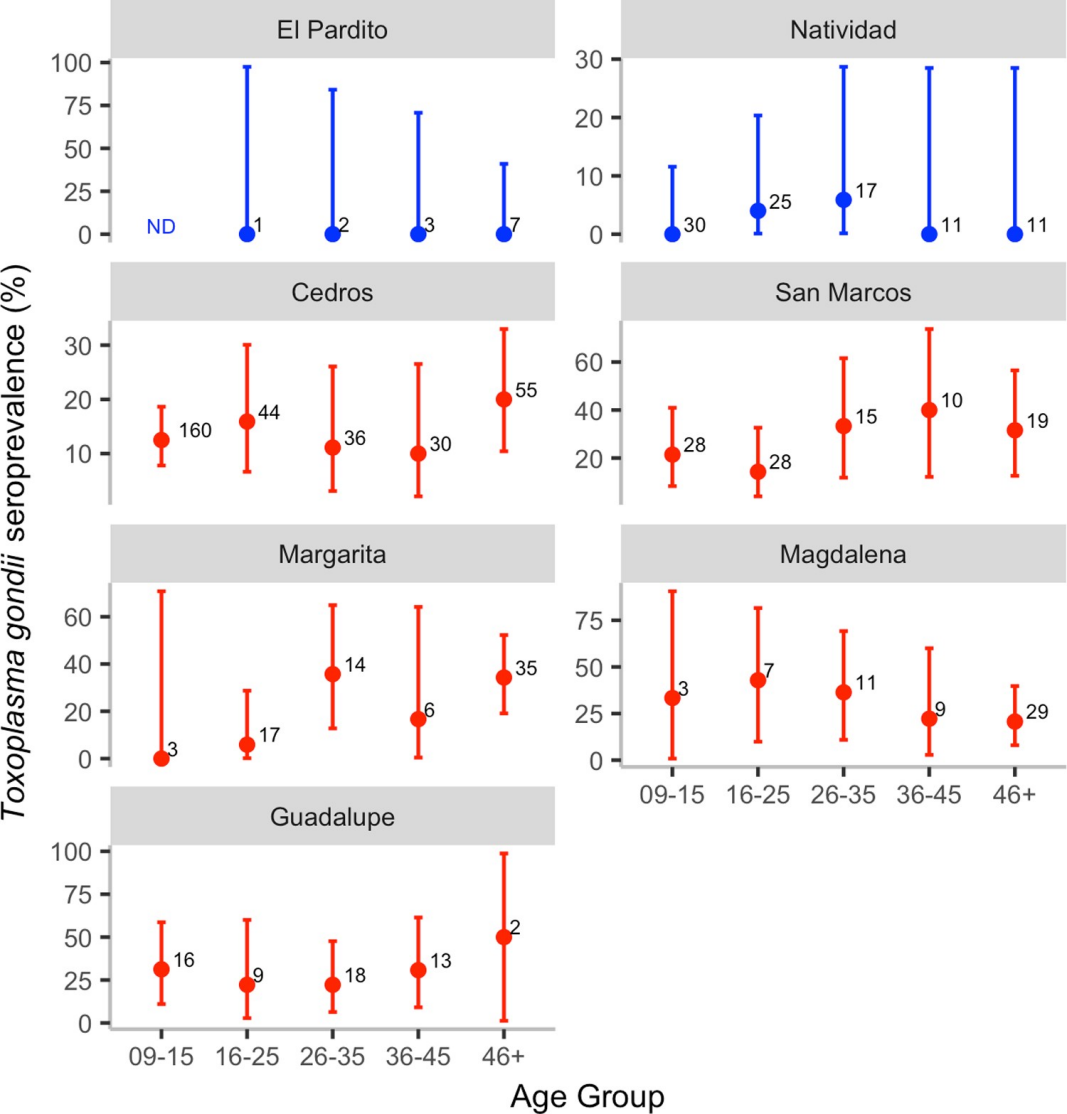
Age-specific *Toxoplasma gondii* seroprevalence. Error bars indicate 95% confidence intervals and numbers indicate sample size. Islands where cats are absent are color-coded blue; islands where cats are present are color-coded red. Children from Natividad in the 09–15 age group were born after cats were eradicated.

We sampled a total of 724 participants of ages 9 to 70 (Table 1 and S2 Appendix). All 13 inhabitants from El Pardito, where cats have never been present, were seronegative for *T*. *gondii* IgG antibodies. The age-adjusted seroprevalence was 1.8% on Natividad, where cats were eradicated in the year 2000. Age-adjusted seroprevalence on the remaining five islands varied from 11.6% to 25.7%, which was significantly higher than on the two cat-free islands (Table 1 and Fig 2).

Seroprevalence varied with age across the islands, with seroprevalence being significantly greater in age groups 26–35, and 46 and older, than 9–16 year olds (Fig 2 and Table 2). The island where cats were eradicated (Natividad) had significantly lower seroprevalence in all age groups compared to most islands with cats. Seroprevalence in the 30 children 9–15 years of age (who were born after cats were eradicated) on Natividad was 0%, which was significantly lower than in children of the same age from three other islands (Fisher’s exact tests: Cedros: 12.5% (20/160), P = 0.04; Guadalupe: 31.2% (5/16), P = 0.01; San Marcos: 21.4% (6/28), P = 0.03) but not in the two others where sample sizes were very small (Margarita: 0% (0/3), P = 1; Magdalena: 33.3% (1/3), P = 0.13). In addition, seroprevalence in people born when cats were still present on Natividad (16 years and older), was significantly lower (3.1%, 2/64, 95% CI = 0.3–10.8%) than that of people of the same age from islands with cats (21.87%, 89/407, 95% CI = 17.9–26.2%).

**Table 2:** Risk factors for *Toxoplasma gondii* seropositivity using a generalized linear mixed effects model with a binomial distribution, a logit link and with island as a random effect. Asterisks indicate the reference level for each predictor.

Risk factor	OR (95% CI)	P value
**Age class**		
9–15*	1	
16–25	1.20 (0.58–2.50)	0.61
26–35	2.03 (1.01–4.10)	0.04
36–45	1.49 (0.73–3.06)	0.26
> 46	1.88 (1.10–3.36)	0.03
**Educational level**	0.88 (0.70–1.11)	0.27
**Gender**		
Female*	1	
Male	1.57 (1.03–2.40)	0.03
**Presence of cats near household**		
No*	1	
Yes	4.79 (2.30–9.97)	< 0.001
**Dog range**		
No dog*	1	
Indoor-outdoor	0.88 (0.53–1.46)	0.61
Outdoor only	1.13 (0.63–2.01)	0.67
**Frequency of shellfish consumption**	0.97 (0.95–0.99)	0.005
**Frequency of meat consumption**	1.01 (0.99–1.02)	0.36
**Raw meat consumption**		
No*	1	
Yes	0.85 (0.42–1.71)	0.64
**Proportion of time spent outside the island**	1.03 (1.01–1.06)	0.02
**Outdoor activities**		
No*	1	
Yes	1.13 (0.67–1.90)	0.64

**Fig 3:**
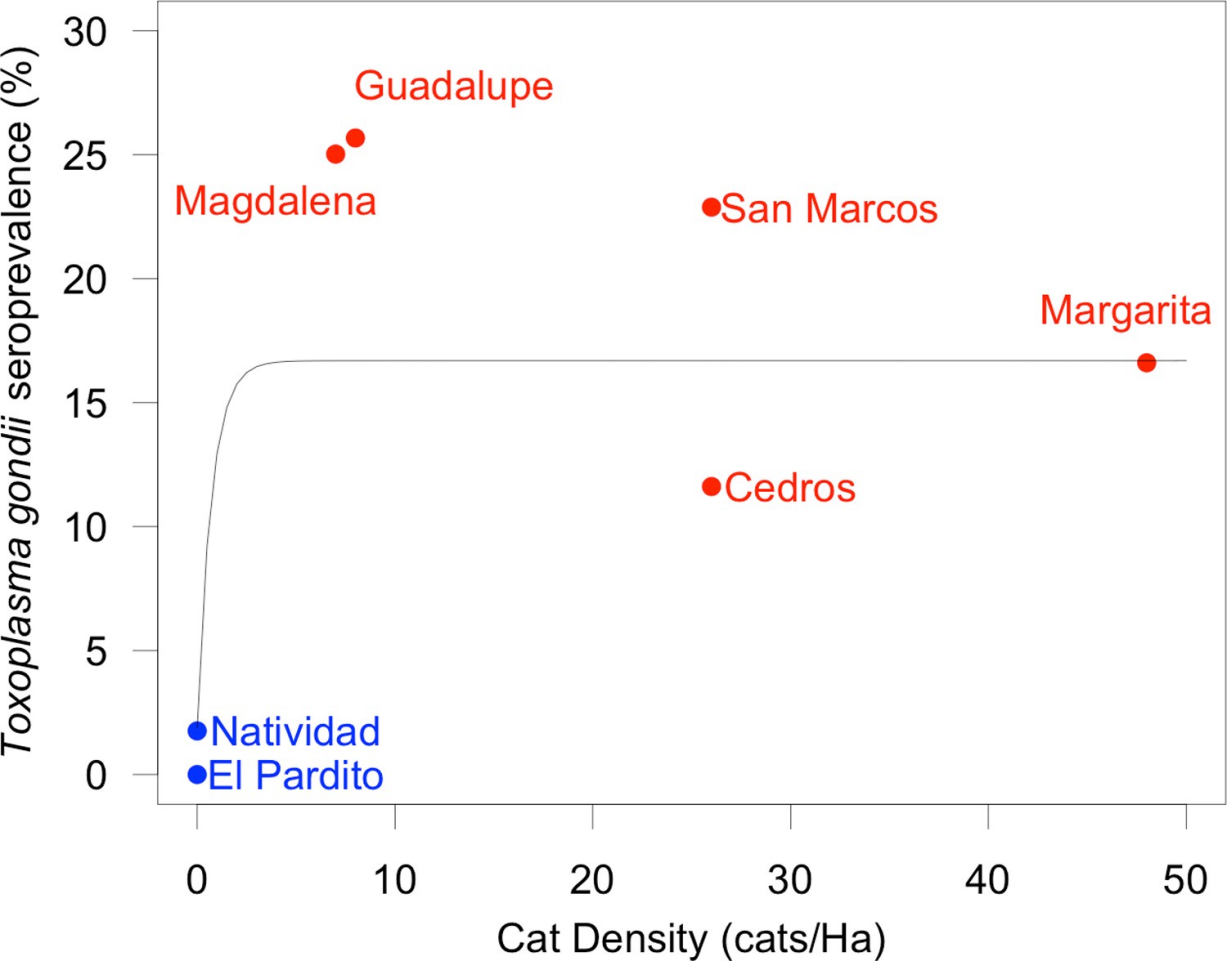
Age-adjusted *Toxoplasma gondii* seroprevalence plotted against the density of cats on seven islands in Baja California. Islands where cats are absent are color-coded blue; islands where cats are present are color-coded red. The best fitting model was Seroprevalence = 0.019+0.15*(1-e^(-1.37*Cat Density)^); slope coefficient P = 0.05; 95% CI: 0.001 –∞).

### Risk factors

We found that interactions with cats, gender, visitation to the mainland, and diet were important factors influencing *T*. *gondii* seroprevalence. The odds of seropositivity to *T*. *gondii* were 4.8-fold higher in people that had cats near their homes. For the subset of people that reported having cats near their homes, *T*. *gondii* seroprevalence decreased with the number of cats reported (S4 and S5 Appendices). The odds of seropositivity were 1.6-fold higher in men (Table 2), 1.03-fold higher for every percent increase of time spent on the mainland, and 0.97-fold lower for every percent increase of shellfish consumption (Table 2). The fitted model indicated that seroprevalence of *T*. *gondii* was 4.3% (1.7% - 6.4%) higher in men than women for the most common age classes of each island (S6 Appendix).

### Cat density

*T*. *gondii* seroprevalence increased with cat density, but the best fitting model showed a sharp rise in seroprevalence between zero cats and the lowest non-zero cat density island, and no change thereafter (Fig 3).

## Discussion

We found evidence that introduced cats are a key source of human exposure to *T*. *gondii* on islands and that eradication, but not control, of cats can reduce the burden of this zoonotic disease. Seroprevalence of *T*. *gondii* was near or equal to zero and significantly lower on the two islands where cats were absent. Moreover, children born after cat eradication were all seronegative. Further, we found that the odds of being seropositive for *T*. *gondii* were nearly fivefold greater for people that reported having cats near their households. However, we found seroprevalence did not increase with overall cat density on islands with cats, and we were surprised to find that for the subset of people that reported having cats near their homes, risk of seropositivity to *T*. *gondii* actually decreased with number of cats reported. Although our findings are based on a relatively small number of study populations from a single geographical region, our focus on island populations (including an island where cats were eradicated) allowed us to examine the effect of cat presence and density, exposure routes, and age more easily than in mainland populations.

Our results suggest that exposure to *T*. *gondii* occurs both in children and in young adults, but may have occurred through different exposure routes. Seroprevalence was lowest in 9–15 year-olds (the youngest group we sampled) with a predicted seroprevalence across all islands of 14%, which increased to 17.0%, 25.8%, 20.3%, and 24.3% in the next four age classes. Studies have suggested that exposure to *T*. *gondii* in children occurs via exposure to oocysts when children play with soil that is contaminated with *T*. *gondii* [18,54,55]. Exposure of young adults may occur through ingestion of bradyzoite cysts in raw or undercooked meat and contaminated produce, likely imported from the mainland [18,55]. To better understand the main sources of *T*. *gondii* infection would require the use of serological tests designed specifically for detecting antibodies against *T*. *gondii* oocysts (e.g. [56]). Finally, we also found older individuals from the island where cats were eradicated (Natividad) to be seronegative to *T*. *gondii*, which may suggest that *T*. *gondii* antibodies wane without antigenic stimulation, as has been suggested elsewhere [57].

Gender, diet, and travel also influenced the risk of *T*. *gondii* exposure. Men had higher risk of *T*. *gondii* seropositivity than women, suggesting that men may engage in activities that increase risk of exposure to *T*. *gondii* (S6 Appendix). We also found that spending time on the mainland was an important risk factor for being exposed to *T*. *gondii*. People may be exposed to contaminated soil, meat or vegetables when traveling to the mainland. The majority of people (83.3%) reported travelling to the northwestern states of Mexico, where the average prevalence of *T*. *gondii* is 39.9% ± 12.9 (Range 20–59.9%) [58]. In contrast, eating shellfish was associated with reduced *T*. *gondii* seroprevalence. How shellfish consumption reduces *T*. *gondii* seroprevalence is unknown, because consumption of shellfish was positively (r = 0.23; N = 724; P < 0.001), not negatively correlated with meat, poultry and pork consumption (potential sources of *T*. *gondii* [10,58,59]).

Our finding that *T*. *gondii* seroprevalence decreased with the number of cats reported for people with cats near their homes suggests that greater cat abundance reduces *T*. *gondii* transmission. While this may seem counterintuitive, higher numbers of cats near a home could actually decrease rodent populations through predation as well as impose sub-lethal effects on rodents through fear and lower fecundity [60]. Reduced rodent density could reduce exposure of *T*. *gondii* in cats by interrupting the predator-prey transmission route of the parasite [9]. Determining whether increased cat abundance reduces transmission of *T*. *gondii* by reducing rodent abundance would require measuring *T*. *gondii* shedding (or at least seroprevalence) in cats as well as rodent abundance across a range of cat densities. Regardless, management or eradication of cat populations should also incorporate management of rodent populations to avoid an increase in rodents as cat predators are removed [61,62]. Increases in rodent population could lead to increasing outbreaks of rodent-borne diseases as well as increased rodent predation on native species [61,62].

Interestingly, we found no increase in seroprevalence with cat density among islands where there were cats. Initially, this appears to contrast with several studies that have found higher *T*. *gondii* seroprevalence in pigs, or humans or *T*. *gondii* in soil in areas with “high” cat density than “low” cat density, or nearer to farms with cat populations than farther away [31–35,37–39]. However, none of these studies examined seroprevalence across a continuous range of cat densities (all treated cat abundance as a categorical variable), and the combination of multiple *T*. *gondii* exposure routes (e.g. soil, food, direct contact with cats) makes studies of human exposure critical. As a result, the actual relationship between cat density and *T*. *gondii* transmission to humans is very poorly understood. The lack of a relationship between cat density and *T*. *gondii* seroprevalence on islands with cats could result from focal aggregation of *T*. *gondii*-contamination in common latrine areas [32,35,63] where cats defecate, but only limited *T*. *gondii*-contamination outside these areas. Spatial sampling of *T*. *gondii* in soil at sites across a range of cat densities would provide data to test this hypothesis. In addition, our estimates of cat density came from a single point in time, and cat densities have likely varied over time. Temporal variation in cat density would make it more difficult to detect a relationship between cat density and seroprevalence. Likewise, the demographic structure of cat populations, which can also vary temporally and as a result of cat population control [62], may also influence transmission dynamics among cats and subsequently exposure to people. This is because kittens lose maternal antibodies against *T*. *gondii* after being weaned and as they begin to consume potentially infected intermediate hosts they are more likely to become infected and shed *T*. *gondii* oocysts [9,64].

Overall, our results suggest that there are opportunities to achieve measurable public health benefits from cat eradications on islands. In contrast, we found little evidence to indicate that controlling cat abundance on islands is an effective tool to reduce human *T*. *gondii* exposure. It remains to be determined how *T*. *gondii* transmission to humans varies with cat density in continental populations, and whether control of feral cat colonies will result in public health benefits without complete or near eradication. Regardless, eradicating zoonotic diseases such as *T*. *gondii* by eliminating their introduced reservoir hosts is much more feasible on islands than in continental populations, and eradicating introduced cats from islands contributes to a “One Health” approach in that this intervention simultaneously benefits human health and native biodiversity [42,65,66].

## Supporting information

S1 Appendix Questionnaire adapted to the social context of communities living in the islands of Baja California, Mexico (Original version in Spanish). (DOCX)

S2 Appendix Demographic characteristics of the sampled population and estimated density of introduced cats in the islands of Baja California, Mexico. (DOCX)

S3 Appendix Feral cat density estimates for the human inhabited islands of Baja California, Mexico. (DOCX)

S4 Appendix Risk factors for *T*. *gondii* seropositivity for the subset of people reporting having cats near their households. Based on a generalized linear mixed effects model with island as a random effect. Asterisks indicate the reference level for each predictor.
(DOCX)

S5 Appendix Association between number of cats reported for people with cats near their homes and seroprevalence of *Toxoplasma gondii*. (TIF)

S6 Appendix Fitted seroprevalence of *Toxoplasma gondii* in men and women of the most common age group from the seven human-inhabited islands of Baja California, Mexico. (TIF)

S7 Appendix Raw data for analysis of seroprevalence and risk factors for *Toxoplasma gondii* infection in the seven human inhabited islands of Baja California, Mexico. (XLSX)
