# Supplementary material for: Potential public health benefits from cat eradications on islands
Source: PLoS Negl Trop Dis. 2019 Feb 14;13(2):e0007040. doi: 10.1371/journal.pntd.0007040 (PMC6392314; doi:10.1371/journal.pntd.0007040)
Supplement: S1 Appendix — (DOCX) [file pntd.0007040.s001.docx]

**S1 Appendix.** Questionnaire adapted to the social context of communities living in the islands of Baja California, Mexico (Original version in Spanish).

1. Gender

__ Female

__ Male

__ Prefers not to answer

1. Year of birth

__

1. Resides in

__ Guadalupe

__ Magdalena

__ El Pardito

__ Margarita

__ Cedros

__ Natividad

__ San Marcos

1. What degree of education do you have?

__ Primary School

__ Middle School

__ High School

__ College degree

__ Graduate degree

__ None of the above

1. Do you have cats in your house?

__ Yes

__ No

1. Do you have indoor, indoor-outdoor or outdoor cats?

__ Indoor

__ Indoor-outdoor

__ Outdoor

1. How many cats do you have in your house (inside or outside)?

__ 1-3

__ 4-6

__ 7-10

__ More than 10

1. What do the cats eat?

__ Dry food/ cat food

__ Leftovers from the house

__ He/she does not feed the cats

1. Do you pick up cat litter?

__ Yes

__ No

1. Do you have dogs in your house?

__ Yes

__ No

1. Do you have indoor, indoor-outdoor or outdoor dogs?

__ Indoor

__ Indoor-outdoor

__ Outdoor

1. Do you spend time in your backyard?

__ Yes

__ No

1. Do you eat raw, undercooked or well-cooked meat (includes beef, pork, chicken and cold cuts)?

__ Raw

__ Undercooked

__ Well-cooked

1. How frequently do you eat meat (includes beef, pork, chicken and cold cuts)?

__ Daily

__ Once a week

__ Once a month

__ Never

14. Do you eat shellfish (includes oysters, clams, abalone, snails)?

__ Yes

__ No

1. How frequently do you eat shellfish?

__ Daily

__ Once a week

__ Once a month

__ Never

1. From where do you get your drinking water?

__ Drinks bottled water

__ Drinks water from the tap

__ Drinks water from the tap but boils it first

__ Desalt plant

1. Do you travel outside of the island?

__ Yes

__ No

1. How often do you travel?

__ Once a week

__ Once a month

__ Once every three months

__ Twice per year

__ Once per year

__ Less than once per year

__ Never

1. How much time do you spend outside of the island you live in?

__ One day

__ One week

__ One month

__ Three months

__ One year

__ Two years

1. Where do you travel most frequently?

__
