# Supplementary material for: Potential public health benefits from cat eradications on islands
Source: PLoS Negl Trop Dis. 2019 Feb 14;13(2):e0007040. doi: 10.1371/journal.pntd.0007040 (PMC6392314; doi:10.1371/journal.pntd.0007040)
Supplement: S2 Appendix — (DOCX) [file pntd.0007040.s002.docx]

**S2 Appendix.** Demographic characteristics of the sampled population and estimated density of introduced cats in the islands of Baja California, Mexico.

| **Island** | **El Pardito** | **Natividad** | **Cedros** | **San Marcos** | **Margarita** | **Magdalena** | **Guadalupe** |
| --- | --- | --- | --- | --- | --- | --- | --- |
| **Total Population*** | 13 | 302 | 1339 | 394 | 156 | 122 | 77 |
| **Sampled population** | 13 | 94 | 325 | 100 | 75 | 59 | 58 |
| **Age sampled**  9-15  16-25  26-35  36-45  46+ | 0  1  2  3  7 | 30  25  17  11  11 | 160  44  36  30  55 | 28  28  15  10  19 | 3  17  14  6  35 | 3  7  11  9  29 | 16  9  18  13  2 |

*** 2015 National census**(1)

**Reference**

1. INEGI. Instituto Nacional de Estadistica y Geografia [Internet]. 2015 [cited 2015 Jan 7]. Available from: http://www.inegi.org.mx/
