# Supplementary material for: Potential public health benefits from cat eradications on islands
Source: PLoS Negl Trop Dis. 2019 Feb 14;13(2):e0007040. doi: 10.1371/journal.pntd.0007040 (PMC6392314; doi:10.1371/journal.pntd.0007040)
Supplement: S3 Appendix — (DOCX) [file pntd.0007040.s003.docx]

**S3 Appendix.** Feral cat density estimates for the human inhabited islands of Baja California, Mexico.

| **Island** | **No. Transects** | **Mean transect length in meters (±SD)** | **Total cat count** | **Density estimate**  **(cats/ha) (±SE)** | **Detection function* (p value**)** |
| --- | --- | --- | --- | --- | --- |
| Cedros | 7 | 416 (77) | 62 | 26 (7) | Hazard-rate (0.41) |
| San Marcos | 3 | 433 (53) | 21 | 26 (5) | Hazard-rate (0.18) |
| Magdalena | 2 | 425 (202) | 10 | 7 (2) | Half-normal (0.47) |
| Margarita | 3 | 178 (56) | 36 | 48 (9) | Hazard-rate (0.32) |
| Guadalupe | 2 | 378 (17) | 7 | 8 (5) | Hazard-rate (0.83) |

**SD =** standard deviation; **SE =** standard error

* Selection of detection function based on smallest AICc value

** Cramer-von Mises test, where a non-significant result suggests that the model fits the data well^1^

**Reference**

1. Miller DL, Rexstad E, Thomas L, Marshall L, Laake J. Distance Sampling in R. bioRxiv [Internet]. 2016;063891. Available from: http://biorxiv.org/lookup/doi/10.1101/063891
