# Supplementary material for: Potential public health benefits from cat eradications on islands
Source: PLoS Negl Trop Dis. 2019 Feb 14;13(2):e0007040. doi: 10.1371/journal.pntd.0007040 (PMC6392314; doi:10.1371/journal.pntd.0007040)

**S4 Appendix.** Risk factors for *T. gondii* seropositivity for the subset of people reporting having cats near their households. Based on a generalized linear mixed effects model with island as a random effect. Asterisks indicate the reference level for each predictor.

| **Risk factor** | **OR (95% CI)** | **P value** |
| --- | --- | --- |
| Age class  9-15*  16-25  26-35  36-45  > 46  Education  Gender  Female  Male  Number of cats reported near household  Contact with cat feces  No  Yes  Dog range  No dog*  Indoor-outdoor  Outdoor  Frequency of shellfish consumption  Frequency of meat consumption  Raw meat consumption  No*  Yes  Proportion of time spent outside the island  Outdoor activities  No*  Yes | 1  1.07 (0.48, 2.37)  1.78 (0.83, 3.83)  1.49 (0.69, 3.24)  2.07 (1.07, 3.98)  0.82 (0.63, 1.05)  1  1.93 (1.22, 3.06)  0.91 (0.84, 0.99)  1  1.11 (0.65, 1.89)  1  0.77 (0.44, 1.33)  0.94 (0.48, 1.84)  0.97 (0.95, 0.99)  1.01 (0.99, 1.03)  1  0.84 (0.40, 1.78)  1.03 (0.99, 1.06)  1  1.09 (0.62, 1.92) | 0.87  0.13  0.30  0.03  0.10  0.004  0.04  0.69  0.34  0.85  0.004  0.31  0.65  0.10  0.74 |

Association between reported cat numbers outside households and *T. gondii* seroprevalence*.* Error bars indicate 95% Confidence Intervals.


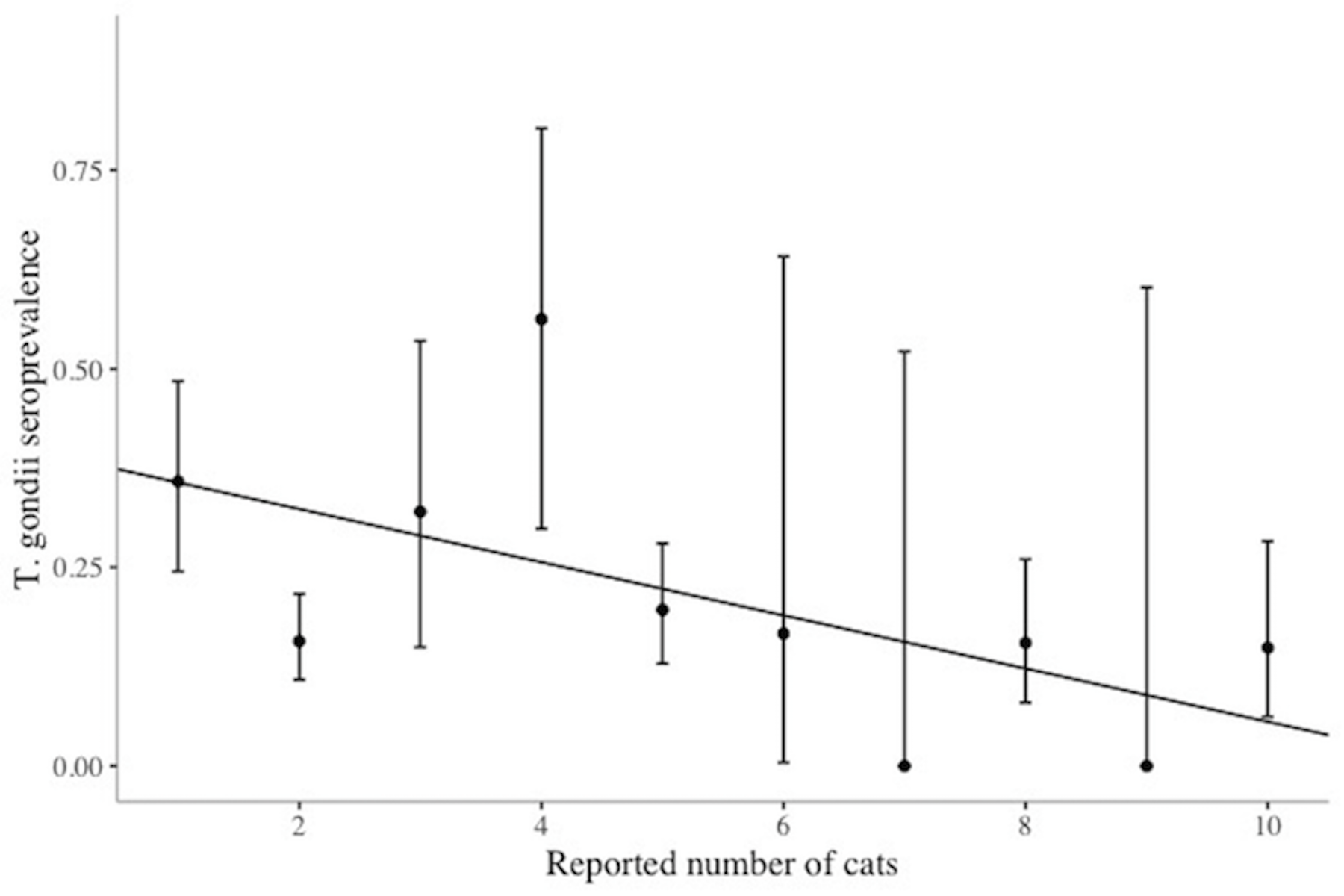

Supplement: S4 Appendix — Based on a generalized linear mixed effects model with island as a random effect. Asterisks indicate the reference level for each predictor. (DOCX) [file pntd.0007040.s004.docx]
