# Supplementary figures and images for: Potential public health benefits from cat eradications on islands
Source: PLoS Negl Trop Dis. 2019 Feb 14;13(2):e0007040. doi: 10.1371/journal.pntd.0007040 (PMC6392314; doi:10.1371/journal.pntd.0007040)

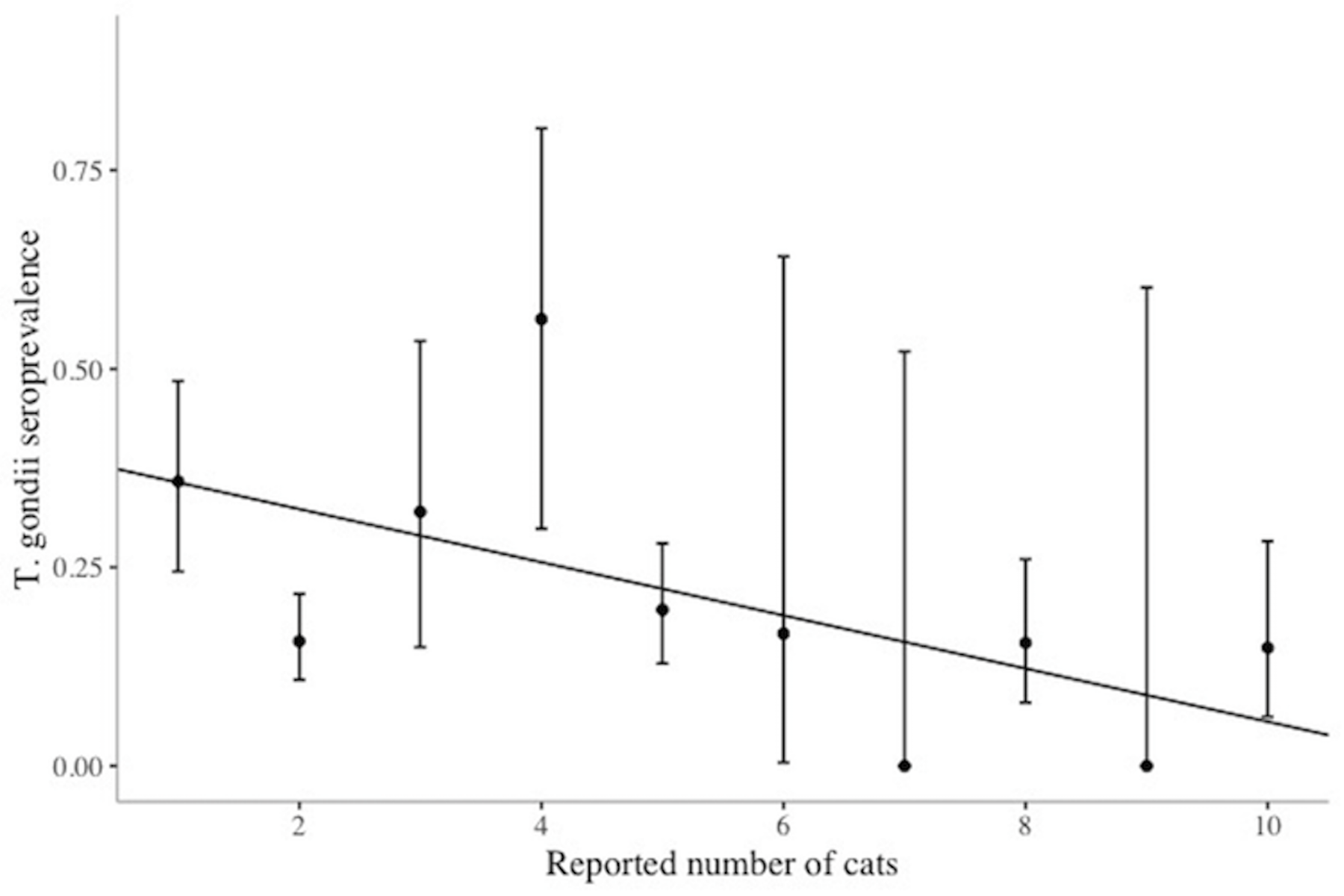

Supplement: S5 Appendix — (TIF) [file pntd.0007040.s005.tif]

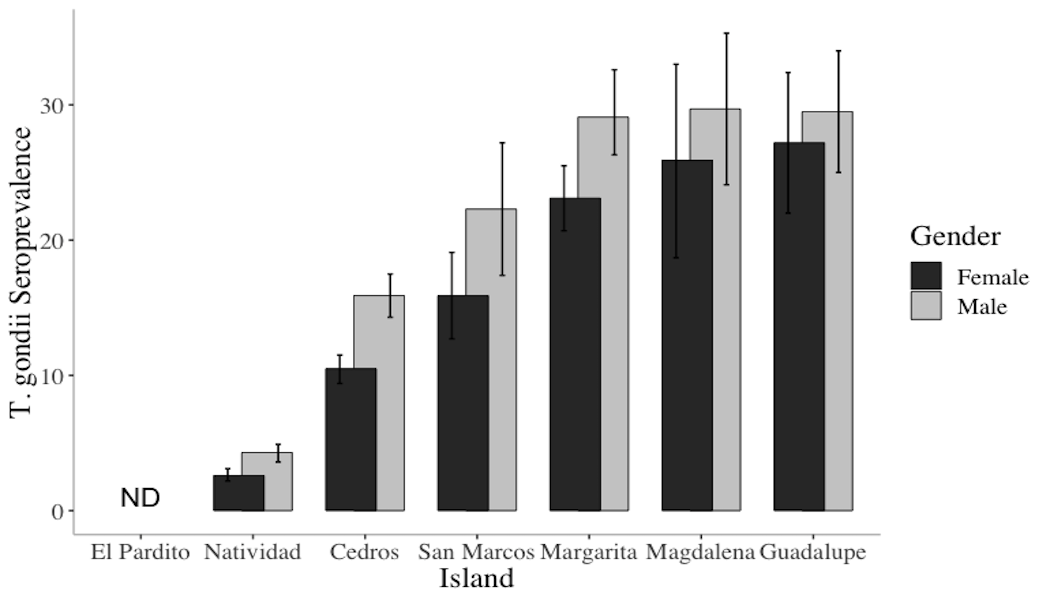

Supplement: S6 Appendix — (TIF) [file pntd.0007040.s006.tif]
